# Concurrent and Convergent Validity of the Simple Lifestyle Indicator Questionnaire

**DOI:** 10.5402/2013/529645

**Published:** 2013-06-01

**Authors:** Marshall Godwin, Andrea Pike, Cheri Bethune, Allison Kirby, Adam Pike

**Affiliations:** Primary Healthcare Research Unit, Discipline of Family Medicine, Memorial University of Newfoundland, St. John's, NL, Canada A1B 3V6

## Abstract

Lifestyle issues including physical activity, diet, smoking, alcohol consumption, and self-reported stress have all been shown to predispose people to higher risk of cardiovascular disease. This study provides further psychometrics on the Simple Lifestyle Indicator Questionnaire (SLIQ), a short, easy-to-use instrument which measures all these lifestyle characteristics as a single construct. One hundred and ninety-three individuals from St. John's, Newfoundland, and Labrador, Canada completed the SLIQ and reference standards for diet, exercise, stress, and alcohol consumption. The reference standards were a detailed Diet History Questionnaire (DHQ), the Social Readjustment Rating Scale (SRRS), the SF36 Health Status Questionnaire, and a survey of eight questions from a cardiovascular risk questionnaire. Physical activity score was compared with number of steps on a pedometer. Correlations between scores on the SLIQ and the reference standards were the SLIQ versus DHQ (*r* = 0.679, *P* = 0.001), SLIQ versus pedometer (*r* = 0.455, *P* = 0.002), SLIQ versus alcohol consumption (*r* = 0.665, *P* = 0.001), SLIQ versus SRRS (*r* = −0.264, *P* = 0.001), SLIQ versus eight-question risk score (*r* = 0.475, *P* = 0.001), and SLIQ versus Question 1 on SF36 (*r* = 0.303, *P* = 0.001). The SLIQ is sufficiently valid when compared to reference standards to be useful as a brief assessment of an individual's cardiovascular lifestyle in research and clinical settings.

## 1. Introduction

Lifestyle issues including physical activity [1, 2], diet [3–5], smoking [6], alcohol consumption [7], and self-reported stress [8] have all been shown to predispose people to higher risk of cardiovascular disease [9].

The Simple Lifestyle Indicator Questionnaire (SLIQ) remains the only short, easy-to-use instrument available for measuring cardiovascular lifestyle as a single construct. It measures five lifestyle risk factors and provides a score for each component, as well as an overall lifestyle score. Initial psychometric testing of its reliability, internal validity, and basic external validity testing was published in 2008 [10]. More details on the SLIQ are in Section 2.

There was still a need to further assess the validity of the individual components. In 2010, with funding through the Healthy Aging Research Program of the Newfoundland and Labrador Centre for Applied Health Research, we conducted further validity testing. This paper reports on the concurrent validity of the physical activity, diet, alcohol, and stress components of the SLIQ. We also report on convergent validity of the whole instrument and compare the overall SLIQ scores to scores on a similar series of questions that have been correlated with cardiovascular morbidity and morbidity.

## 2. Methods

### 2.1. Study Population

We recruited patients aged 40 and above from three different family physician clinics that are part of a practice-based research network and have participated in research studies previously. The practices have electronic medical records (EMRs). Randomly generated lists of patients in three age groups, 40–59 years, 60–79 years, and 80+ years, were compiled by the clinic staff. Initially 100 people in each group were contacted by letter. The letters were printed on physician's letterhead and signed by the physicians. The letters briefly explained the study and provided the phone number of the research assistant to be contacted if they were interested in taking part. Subsequently two further blocks of 100 people per age group were contacted until we had sufficient sample size. We sent letters to 900 people in order to recruit 193 consenting patients who completed all the questionnaires.

### 2.2. Sample Size Requirements

Using an alpha of 0.05 and a beta of 0.2, we calculated that 153 participants would be needed to make sure that a correlation of 0.2 or more was statistically different from a correlation of zero. Therefore, our sample size of 193 provided sufficient power since all our correlations turned out to be greater than 0.2.

### 2.3. Reference Standards

Each participant provided basic demographic data and completed the SLIQ instrument, a detailed Diet History Questionnaire (DHQ) [11, 12], the Social Readjustment Rating Scale (SRRS) [13, 14], Question 1 on the SF36 Health Status Questionnaire [15–18], and a survey of eight questions on cardiovascular risk proposed by Spencer et al. [19]. Moreover, each participant wore a pedometer for three days and returned the pedometer to the research assistant.

Using a pedometer for three days has been shown to be highly correlated with the level of physical activity [20, 21]. The pedometer used, the Sportline SL330, has been shown to be equivalent to a reference pedometer which measures within 3% of the actual number of steps [21].

The DHQ collects detailed information on type and quantity of different foods (vegetables, fruit, grains, meat, fish, dairy products, etc.) eaten on a weekly basis over the previous year. It also collects detailed information on alcohol consumption [11, 12].

The SRRS collects information on stressful life events in the past year. It is a validated instrument which scores 43 life events from 12 to 100. The higher the score the higher the likelihood of experiencing stress [13, 14].

The SF36 is a health status measurement tool used in over 4000 published studies worldwide. Its psychometrics has been assessed in all age groups, genders, and many races and countries. Question 1 on the SF36 is used as a measure of self-assessed health status [15–18]. Question 1 asks “In general, would you say your health is □ Excellent □ Very Good □ Good □ Fair □ Poor.”

In 2005, Spencer et al. published a study completed in Australia on healthy elderly men. They compared the response to eight questions on diet, activity, smoking, alcohol, and BMI to a person's likelihood of dying. People with a score of 5 out of 8 or higher had a 5 times higher likelihood of dying within 5 years. Psychometric properties of this scale have not been reported. We will compare the SLIQ lifestyle score to the score on this eight-question scale [19].

Concurrent validity is determined by comparing the score on the instrument of interest; in this case the SLIQ, with the score on a reference standard—a measurement tool that is known to accurately measure that same construct. Concurrent validity in this study is determined for physical activity by comparing the physical activity score on the SLIQ with the steps on a pedometer; it is determined for diet by comparing the raw diet score on the SLIQ with the diet score on the DHQ; it is determined for alcohol by comparing the raw alcohol component on the SLIQ with the alcohol score on the DHQ; and it is determined for stress by comparing the raw stress score on the SLIQ with the stress score on the SRRS. Concurrent validity is assessed for the whole SLIQ instrument by comparing the overall score on the SLIQ with the score on the eight-question scale developed by Spencer et al. For concurrent validity, we hoped to achieve correlation coefficients of 0.4 or higher and ideally larger than 0.6.

Convergent validity is measured by comparing the instrument in question with another instrument that measures a related, but different, construct. In this case, we used the responses to the first question on the SF36 which is a self-assessment of one's health status. While lifestyle and health status are related, they would not be expected to have high correlation since a change in one may precede or follow the other rather than necessarily exist concurrently. We expected a correlation coefficient in the range of 0.2 to 0.4.

### 2.4. The Simple Lifestyle Indicator Questionnaire (SLIQ)

Measuring human behaviour is not an exact science and rarely can it be done with the precision found in fields such as engineering. To increase the accuracy of measurement, we often have to use long and detailed questionnaires or similar meticulous processes. However, what is preferred is a measure that is short and easy, especially in a clinical setting where providers are busy and patients may be ill. Similarly, in a health research setting the issue of participant burden has to be considered and long detailed questionnaires should be avoided. In order to efficiently measure some behaviours in clinical and research settings, shorter health measurement scales are developed and compared to the longer detailed assessments to determine if they can be used to realistically assess the particular behaviour or health issue. This was the motive behind the development of the SLIQ. The original development process started eight years ago with over 30 questions, and, using factor analysis, the number of questions was decreased to twelve. It is this 12-question SLIQ that was tested in the initial psychometrics study published in 2008 [10] and which was used in this current study.

The SLIQ has 12 questions; three on diet, three on physical activity, three on alcohol consumption, two on smoking, and one on stress—see Supplementary Figure 1 (see Supplementary Materials available online at http://dx.doi.org/10.5402/2013/529645). A French version is also available, but none of the psychometric testing has been carried out in French. It was developed as a short and simple health measurement scale [10]. Each component is assigned a category score of 0, 1, or 2, based on raw scoring of questions related to each component. Component scores are summed to give a SLIQ score from 0 to 10 (0 = very unhealthy, 10 = very healthy). Categorically, a person is considered “unhealthy” if they have a SLIQ score of between 0 and 4, “intermediate” if the SLIQ score is between 5 and 7, and “healthy” if they score between 8 and 10 on the SLIQ. Initial psychometric testing of the SLIQ [10] resulted in a test-retest reliability between 0.63 and 0.97; a Cronbach *α* of 0.58 for diet and 0.6 for physical activity, and blinded external validity of 0.77. The scoring template is shown in Supplementary Figure 2.

### 2.5. Analysis

In this current study, validity was assessed by determining the correlation coefficients between the component raw scores on the SLIQ and the appropriate reference standards (DHQ, SRRS, Question 1 on the SF36, and step per day on the pedometer). ANOVA was conducted to determine if the three categories, unhealthy, intermediate, and healthy, were separately distinguishable populations for each component and for the overall SLIQ score. Scatter plots with best-fit lines are also presented for each component. Finally, we compare the overall SLIQ score with the score on the eight-question risk assessment developed by Spencer et al. [19].

### 2.6. Ethics and Consent

This research was reviewed and approved by the Human Investigation Committee of Memorial University of Newfoundland, Canada. All participants gave their consent before enrolling in the study.

## 3. Results

One hundred and ninety-three adults completed all the questionnaires allowing correlational statistics to be completed. The mean age was 65 years (SD = 14.5 years) with a range 40–95 years; nearly sixty-six percent were females. Details of education, marital status, income, and BMI are shown in Table 1. The population was distributed through the socioeconomic spectrum but was financially better and more educated than the general population. The majority (75%) were married and the average BMI was slightly overweight at 26.5.

**Figure 1:**
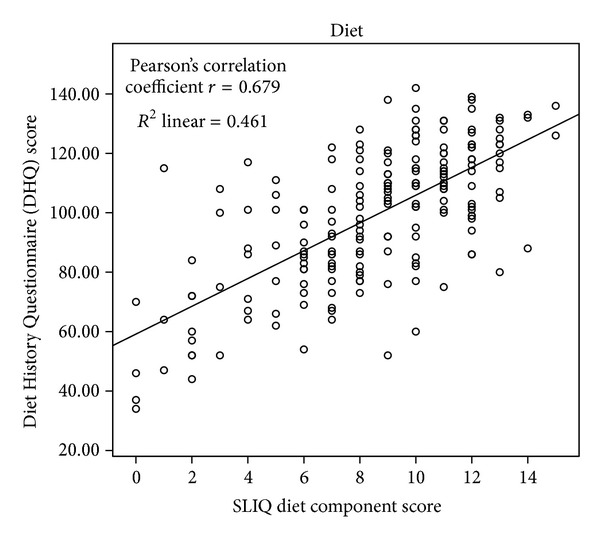
SLIQ versus Diet History Questionnaire concurrent validity.

**Figure 2:**
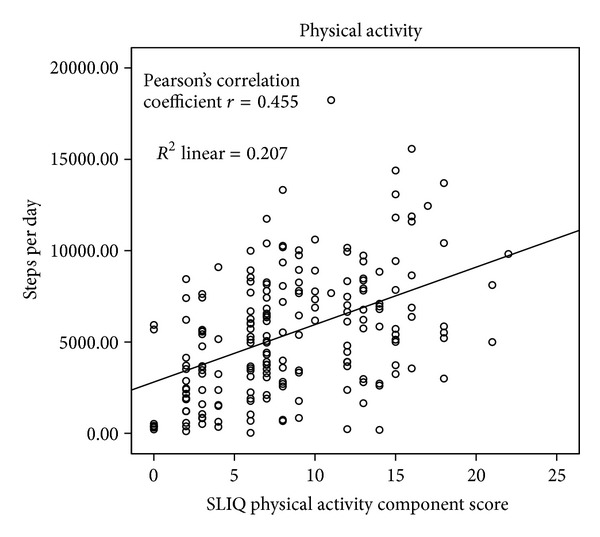
SLIQ versus pedometer concurrent validity.

**Figure 3:**
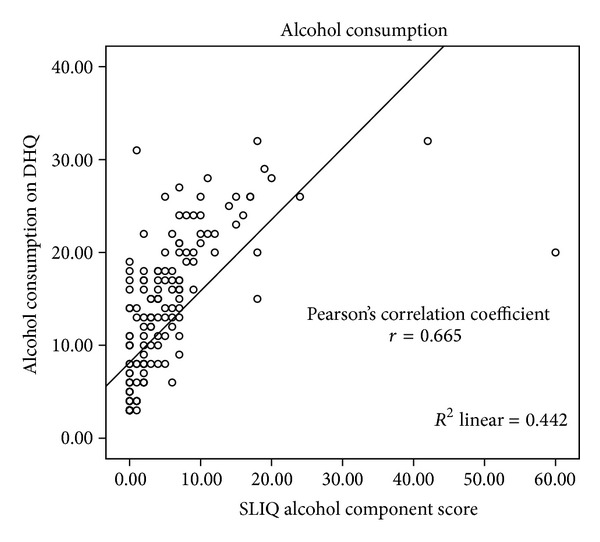
SLIQ versus alcohol consumption concurrent validity.

**Figure 4:**
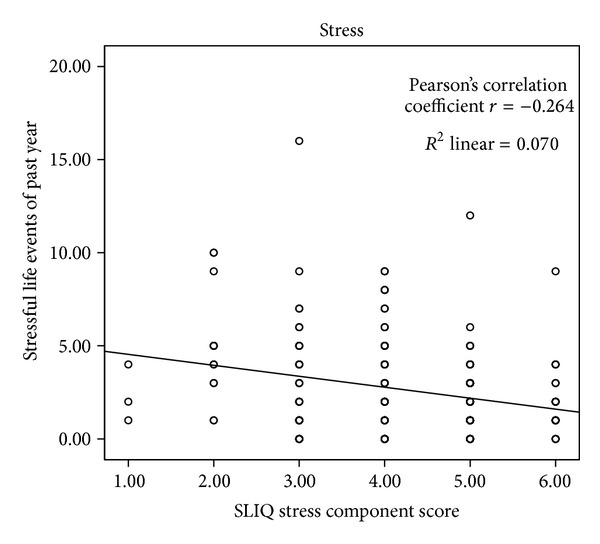
SLIQ versus stressful life events concurrent validity.

**Figure 5:**
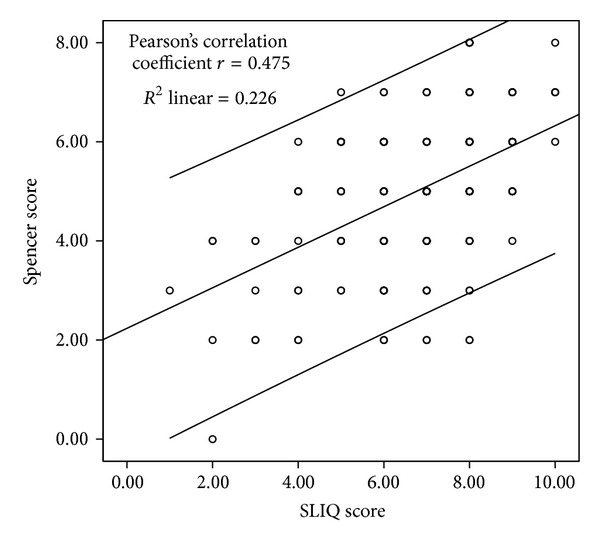
SLIQ lifestyle score versus score of eight questions developed by Spencer et al.

**Figure 6:**
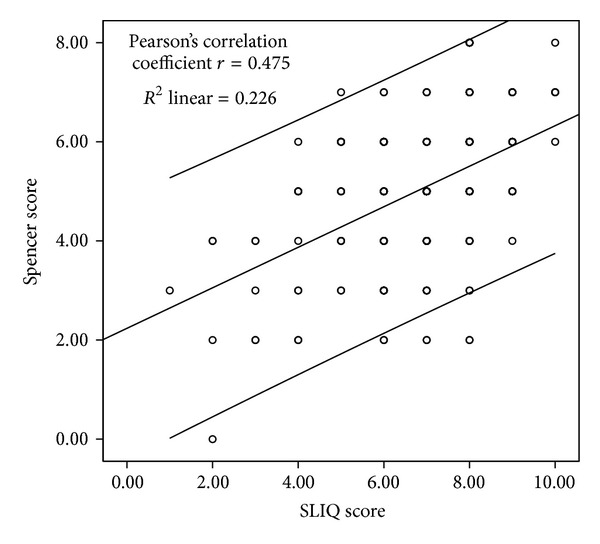
SLIQ lifestyle score versus self-assessed health on SF36.

The details of the correlations are presented in Table 2 and Figures 1, 2, 3, 4, 5, and 6. The SLIQ diet and alcohol components correlate well with diet and alcohol as assessed by DHQ (*r* = 0.679 and 0.665, resp.); there is also good correlation between the SLIQ physical activity component and the number of steps over three days as measured by a pedometer (*r* = 0.455). The SLIQ stress component did not correlate as well as we had hoped with stress as measured by the stressful life events count on the SRRS (*r* = −0.264). The correlation with stressful life events is negative because on the SLIQ a higher score for stress means less stress whereas on the SRSS a higher count indicates more stress. The overall SLIQ lifestyle score correlated reasonable well with the eight-question risk scale (*r* = 0.475), and people who score 5 or higher on the eight-question scale had a mean SLIQ score of 7.2 (SD 1.4) while those who scored less than 5 on the eight questions scale had a mean SLIQ score of 5.8 (SD 1.8); these two means were from statistically different populations, *P* = 0.001. The final comparison, between the SLIQ lifestyle score and self-assessed health on the SF36 (Question 1), is considered an assessment of convergent validity rather than concurrent validity in that the expectation is not that the correlations would be high but rather in the fair or moderate range. This is because lifestyle and health status are not measurements of the same construct but rather related constructs. Hence, a correlation of *r* = 0.303 is in the range we were expecting.

The categorization of individuals as unhealthy/intermediate/healthy was assessed to determine if these three groups were from separate populations; that is, whether the mean and standard deviations of the SLIQ scores in these three groups were separated such that there was very little overlap. We used ANOVA to determine the mean and standard deviations and 95% CI for these groups. The data in Table 3 shows that the three categories are clearly separated with almost no overlap.

## 4. Discussion

The correlation coefficient, usually designated as “*r*”, quantifies the strength of the linear relationship between two variables. An *r* of zero indicates no correlation and an *r* of 1 means a perfect linear relationship. Opinion on how a given level of correlation between 0 and 1 should be interpreted varies in the literature. Some investigators believe a correlation between 0.0 and 0.25 represents a weak relationship, 0.26–0.50 a moderate relationship, 0.51–0.75 a strong relationship, and greater than 0.75 a very strong correlation. Others divide the correlation coefficient into three categories where an *r* between 0.0 and 0.3 indicates a weak relationship, 0.3 to 0.7 indicates a moderate relationship, and >7 indicates a strong relationship [22]. In 2003, Hemphill [23] reviewed the literature that reported correlation coefficients and divided them into tertiles: one-third reported coefficients <0.2, the middle third reported coefficients between 0.2 and 0.3, and the upper third reported coefficients >0.3. And finally Cohen [24] uses a benchmark of *r* = 0.50 as a level of strong correlation. The correlation coefficients reported in this study vary from 0.264 to 0.679. The interpretation systems described above would place most of these *r* values in the moderate-to-strong categories.

As mentioned in the results, the correlation for SLIQ stress score versus the SRRS stress score is negative. This is expected … all SLIQ components are scored such that a higher number means a better lifestyle (in this case lower stress). The SRRS is scored such that a higher number means higher stress. The absolute value of the correlation for the stress component is the lowest of all the components, and we considered dropping it from the scale. However, the SLIQ measures the person's assessment of their current stress level while the SRRS simply scores stress events in a person's life over the previous year. People deal with stress differently, and the person's assessment of their own feelings of stress may be more important.

Validity correlation coefficients for diet, physical activity, and alcohol consumption are sufficiently strong to make the SLIQ useful in research and clinical settings. Similarly, its correlation with the eight-question scale, which themselves have been shown to predict mortality, increases the SLIQ validity.

Assessments of the SLIQ sensitivity to change and its predictive validity are both underway.

*Limitations*. The chosen reference standard, and whether it is the most appropriate, is always an issue with validity studies. The DHQ is a long and detailed questionnaire and is probably as good as any we could have chosen. The SRRS takes an interesting approach to assessing stress in a person's life but one could argue there are more modern approaches, and perhaps we should have chosen one of those. It has been suggested that we should have used an accelerometer to measure actual physical activity rather than a pedometer. Cost was one issue for us in this regard. It has also been suggested that since the eight questions used by Spencer et al. have been shown to predict mortality, why not we use them rather than the SLIQ for assessing lifestyle. One reason is that Spencer included BMI which is not a lifestyle but rather a consequence of lifestyle; moreover, there have been no psychometrics conducted on the eight questions unlike the SLIQ which has a developing literature. Finally, the study population was somewhat skewed towards women (65%) and older age group (mean age 65 years).

## 5. Conclusion

We believe the SLIQ is sufficiently valid when compared to reference standards to be useful as a brief assessment of an individual's cardiovascular lifestyle in research and clinical settings.

## Supplementary Material

Supplementary Figure 1: Shows the 12 questions in the SLIQ with exact wording of the questions and the response options
Supplementary Figure 2: Show the 12 questions on the SLIQ and how raw and category scores are calculated for each of the five component scores and the Overall SLIQ scores.

## Figures and Tables

**Table 1:** Study population demographics.

Age, *n* = 193	
Mean: 65 years; SD: 14.5 years	
Range: 40–99 years	
Gender, *n* = 193	
Female: 127 (65.8%)	
Male: 66 (34.2%)	
Marital status, *n* = 167	
Single	20 (12.0%)
Married	125 (74.9%)
Separated	3 (1.8%)
Divorced	12 (7.2%)
Common law	7 (4.2%)
Income, *n* = 145	
≤$25,000	17 (11.7%)
$25,001–$35,000	18 (12.4%)
$35,001–$50,000	16 (11.0%)
$50,001–$75,000	31 (21.4%)
$75,001–$100,000	25 (17.3%)
$100,001–$150,000	21 (14.5%)
$150,001–$200,000	15 (10.3%)
>$200,000	2 (1.4%)
Education, *n* = 192	
Did not complete high school	21 (10.9%)
Completed high school	25 (13.0%)
Some college or university	30 (15.6%)
Completed college diploma or university degree	42 (21.9%)
Some postgraduate or professional training	26 (13.5%)
Completed postgraduate or professional training	48 (25.0%)
BMI, *n* = 184	
Mean: 26.5; SD 4.8	
Range: (17.5–44.6)	

**Table 2:** Pearson's correlation coefficients of concurrent and convergent validity.

SLIQ component	Validity criterion	Correlation (*ρ*)	*P*value (*α* = 0.05)
Diet	DHQ (vegetables/fruits/grains)	0.679	0.001
Physical activity	Pedometer (average steps/day)	0.455	0.002
Alcohol	DHQ (alcohol)	0.665	0.001
Stress	SRRS	−0.264	0.001
SLIQ lifestyle score	Eight-question scale developed by Spencer et al. [19]	0.475	0.001
SLIQ lifestyle score	Self-assessed health on the SF36 (Question 1)	0.303	0.001

**Table 3:** Comparison of SLIQ scores in the three category levels of unhealthy, intermediate, and healthy using ANOVA.

	Mean SLIQ score	Standard deviation	95% CI	Bonferroni *P* values between all groups
Unhealthy, *n* = 20	3.2	0.95	2.7–3.6	0.001
Intermediate, *n* = 104	6.3	0.73	6.2–6.5	
Healthy, *n* = 69	8.4	0.62	8.2–8.5	
