# Supplementary material for: Concurrent and Convergent Validity of the Simple Lifestyle Indicator Questionnaire
Source: ISRN Family Med. 2013 Jun 1;2013:529645. doi: 10.5402/2013/529645 (PMC4041224; doi:10.5402/2013/529645)
Supplement: Supplementary file 1 — Supplementary Figure 1: Shows the 12 questions in the SLIQ with exact wording of the questions and the response options Supplementary Figure 2: Show the 12 questions on the SLIQ and how raw and category scores are calculated for each of the five component scores and the Overall SLIQ scores. [file 529645.f1.pdf]

Figure 1: Simple Lifestyle Indicator Questionnaire

Diet: To answer these questions, think about your eating habits in the past year. Indicate how often you eat the following foods. Please include all meals, snacks and eating out

1. Lettuce or green leafy salad, with or without other vegetables
   [ ] less than 1/week [ ] 1/week [ ]2-3x/week [ ]4-6x/week [ ] once/day [ ] 2+/day
2. Fruit: include fresh, canned or frozen, but do not include juices
   [ ] less than 1/week [ ] 1/week [ ]2-3x/week [ ]4-6x/week [ ] once/day [ ] 2+/day
3. High fiber cereals or whole grain breads: this includes cereal such as Raisin bran, Fruit and Fiber, cooked oatmeal, and breads which are whole wheat, multigrain, rye or pumpernickel
   [ ] less than 1/week [ ] 1/week [ ]2-3x/week [ ]4-6x/week [ ] once/day [ ] 2+/day

Physical Activity: To answer the following questions, think about your physical activity over the past year, please indicate on average how many times per week you take part in the following activities for a duration of at least 30 minutes or more at a time:

I. Light physical activity, such as:

- light gardening and light housework (dusting, sweeping, vacuuming)
- leisurely walking (walking your dog)
- bowling, fishing, carpentry, playing a musical instrument
- volunteer work

[ ] 0/week [ ] 1-3x/week [ ] 4-7x/week [ ] 8 and more/week

II. Moderate physical activity, for example:

- brisk walk
- bicycling, skating, swimming, curling
- gardening (raking, weeding, spading)
- dancing, Tai Chi or moderate exercise classes

[ ] 0/week [ ] 1-3x/week [ ] 4-7x/week [ ] 8 and more/week

III. Vigorous physical activity, for example:

- running, bicycling, x-country skiing, lap swimming, aerobics
- heavy yard work
- weight training
- soccer, basketball or other league sports

[ ] 0/week [ ] 1-3x/week [ ] 4-7x/week [ ] 8 and more/week

Alcohol: Please indicate how many drinks of the following types of alcohol you consume in an average week:

- ✓ Wine: _____ drinks (3-5 oz.)
- ✓ Beer: _____ drinks (10-12 oz or 1 bottle)
- ✓ Spirits: ____ drinks (1-1 ½ oz.)

Smoking: Please indicate your smoking habits below:

Are you a smoker? [ ] Yes [ ] No
If yes, how long have you been smoking? __________ years
If no, did you ever smoke? [ ] Yes [ ] No
If yes, how many years ago did you quit? ___________ years

Life Stress: To answer this question please circle the number which you feel best corresponds to the level of stress in your everyday life

1 2 3 4 5 6
Not at all stressful ... Very stressful

Figure: 2 Scoring Template for the SLIQ

Scoring the
Simple Lifestyle Indicator Questionnaire

Diet:

1. Lettuce or green leafy salad, with or without other vegetables					
□less than 1/week<br>0	□ 1/week<br>1	□ 2-3x/week<br>2	□ 4-6x/week<br>3	□ once/day<br>4	□2+/day<br>5
2. Fruit: include fresh, canned or frozen, but do not include juices					
□less than 1/week<br>0	□1/week<br>1	□2-3x/week<br>2	□ 4-6x/week<br>3	□once/day<br>4	□ 2+/day<br>5
3. High fiber cereals or whole grain breads: this includes cereal such as Raisin bran, Fruit and Fiber, cooked oatmeal and breads which are whole wheat, rye or pumpernickel					
□less than 1/week<br>0	□ 1/week<br>1	□2-3x/week<br>2	□ 4-6x/week<br>3	□ once/day<br>4	□2+/day<br>5

DietRawScore = _______
Q1+Q2+Q3

DietCategoryScore = __________
0 If DietScore 0-5
1 If DietScore 6-10
2 If DietScore 11-15

Exercise:

1. Light exercise, such as:
   - light gardening and light housework (dusting, sweeping, vacuuming)
   - leisurely walking (walking your dog)
   - bowling, fishing, carpentry, playing a musical instrument
   - volunteer work

□0/week	□1-3x/week	□4-7x/week	□8 and more/week
0	2	3	4

2. Moderate exercise, for example:
   - brisk walk
   - bicycling, skating, swimming, curling
   - gardening (raking, weeding, spading)
   - dancing, Tai Chi or moderate exercise classes

□0/week	□1-3x/week	□4-7x/week	□8 and more/week
0	4	6	8

3. Vigorous exercise, for example:
   - running, bicycling, x-country skiing, lap swimming, aerobics
   - heavy yard work
   - weight training
   - soccer, basketball or other league sports

□0/week	□1-3x/week	□4-7x/week	□8 and more/week
0	6	9	12

ActivityRawScore = _______
Q1+Q2+Q3

ActivityCategoryScore = _____
0 If Light Exercise Only
1 If Any Moderate Activity
2 If Any Vigorous Activity

Figure 2 continued: Scoring Template for the SLIQ

Alcohol: (per week)

- ✓ Wine: _____ drinks (3-5 oz.)
- ✓ Beer: _____ drinks (10-12 oz or 1 bottle)
- ✓ Spirits: ____ drinks (1-1 ½ oz.)

AlcoholRawScore _____
Wine + Beer + Spirits

AlcoholCategoryScore ________

0 If Alcohol Score 14 or more
1 If Alcohol Score 8-13
2 If Alcohol Score 0-7

Smoking:

Are you a smoker?
☐Yes ☐No
0

If no, did you ever smoke?
☐Yes ☐No
1 2

SmokingRawScore _____
(0,1, or 2)
SmokingCategory Score________
(Same as Smoking Score)

Life Stress:

6 5 4 3 2 1

Not at all stressful ... Very stressful

StressRawScore = _________
As indicated on Line

StressCategoryScore _______
O If Life Stress 1 or 2
1 If Life Stress 3 or 4
2 If Life Stress 5 or 6

SLIQScore = DietCategory + ActivityCategory + AlcoholCategory + SmokingCategory + StressCategory
